# Use of *Ganoderma lucidum* grown on agricultural waste to remove antibiotics from water

**DOI:** 10.1039/d5ra06482a

**Published:** 2026-01-05

**Authors:** Vanessa Juarez, Shiva Emami, Ameer Y. Taha, Valeria La Saponara

**Affiliations:** a Department of Mechanical and Aerospace Engineering, University of California, Davis One Shields Avenue Davis CA 95616 USA vlasaponara@ucdavis.edu; b Department of Food Science and Technology, University of California, Davis One Shields Avenue Davis CA 95616 USA ataha@ucdavis.edu

## Abstract

Antibiotic-rich effluents from farming and medical establishments into waterways pose a serious risk for antibiotics resistance, promoting a need for effective strategies of removal from the food chain and the environment. In this work, we show proof-of-concept laboratory-scale low-cost bioremediation experiments to remove antibiotics in synthetic wastewater. A white rot fungus, *Ganoderma lucidum*, was grown on biomass formed by agricultural waste from California (almond shells, cover crop stalks). Water containing or lacking *Ganoderma lucidum* was inoculated with twenty antibiotics from six different classes. The extent of antibiotic removal was measured at baseline and after 3 days with ultra-high pressure liquid chromatography coupled to tandem mass-spectrometry. The data were analyzed with a two-way repeated ANOVA for 17 antibiotic data sets meeting residuals' normality, and a mixed-effects model for 3 antibiotics that did not. Treatment with mycelial biomass for 3 days caused a statistically significant reduction, compared to the baseline, in the concentration of 3 quinolones and 1 sulfonamide. There were similar non-significant trends or neutral results in the other 16 antibiotics within those 3 days. Within the limitations of our work, our findings provide a first proof-of-concept on the potential to bioremediate certain antibiotics, particularly quinolones and sulfonamides, in synthetic wastewater and with repurposed agricultural waste.

## Abstract 2

Antibiotic-rich effluents from farming and medical establishments into waterways pose a serious risk for antibiotics resistance, promoting a need for effective strategies of removal from the food chain and the environment.

## Graphical abstract

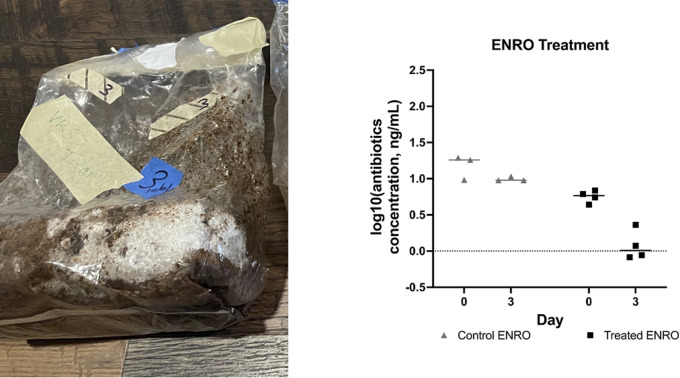

## 1. Introduction

Resistance of bacteria, parasites, viruses and fungi to antimicrobial agents (Antimicrobial Resistance, AMR) is associated with greater risk of difficult-to-treat infections in humans.^1^ It is estimated that AMR caused 4.95 million premature deaths worldwide in 2019.^2^ The first comprehensive assessment of global AMR in the period 1990–2021, published by The Lancet in September 2024, presented also a forecast for 2050, with an estimated total of up to 10 millions annual deaths (directly or indirectly) worldwide due to AMR.^3^ The World Bank sized the economic cost of AMR to be equivalent, in the worst case scenario, to an average annual drop of 3.8% of gross domestic product, or GDP.^4^ The largest burden will be disproportionately carried by low-income countries, with an estimated increase of 18 million people living in extreme poverty in 2050.^4^

Environmental pollution (legacy and emerging, such as heavy metals, polycyclic aromantic compounds, microplastics, *etc.*) impacts AMR: it reduces the diversity of ecosystems' microbiome and decreases the rate of degradation of antibiotics in the environment (*e.g.* through adsorption to microplastics). The interplay of these pollutants with geochemical conditions and synergistic effects on antibiotic-resistant bacteria and genes (ARGs) is an active research topic (see for example ref. 1 and 5–7).

Unsurprisingly, climate change is also exacerbating AMR proliferation in multiple ways. Studies have shown that increasing local minimum temperatures due to climate change increases the risk of AMR.^7–9^ In addition, Fagunwa *et al.*^10^ discussed the challenges of safe pharmaceutical storage in resource-poor countries experiencing extreme environmental conditions due to climate change: in these situations, antimicrobials' potency is reduced, and surviving pathogens are more likely to develop resistance mechanisms.

Antibiotics are used and misused in medical, veterinary, and farming (both aquaculture and agriculture, see for example ref. 11–13), and can enter the environment through a variety of complex ecosystem/environmental interactions, exemplified by Fig. 1 from ref. 1.

**Fig. 1:**
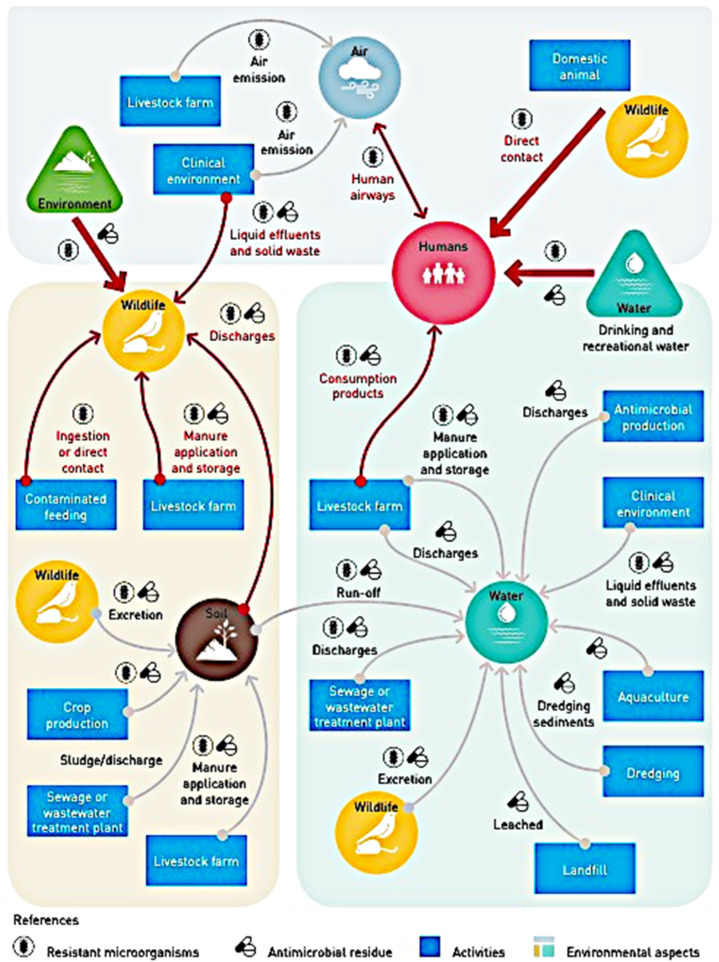
Environmental complexities in transmission and spread of antimicrobial resistance.^1^

Water serves as a vehicle for the evolution and proliferation of ARGs, whether freshwater (a “gene exchange bridge between otherwise disconnected habitats”^14^), or wastewater effluents and agricultural runoffs, *e.g.* ref. 11, 13 and 15–21. Wastewater treatment plants (WWTPs) are typically unable to remove effectively every single antibiotic type. Thus, high antibiotic concentrations (mg mL^−1^) have been found in WWTPs, where AMR bacteria and genes have been detected (*e.g.* ref. 20, 22 and 23). Untreated wastewaters reaching the ocean have in turn caused the ocean to be a reservoir of antibiotics and a “global reservoir of both clinically relevant and potentially novel antibiotic resistant genes”.^24^ Even very low antibiotic concentrations (100 pg mL^−1^ to 15 ng mL^−1^) in aquatic and soil ecosystems could maintain resistant bacteria.^25^

To reduce the spread of AMR-bacteria and genes, the UNEP^1^ report lists worldwide-coordinated “prevention […] at the core of the action and environment […] as a key part of the solution”. When considering the removal of antibiotics from wastewater, methods include activated carbon adsorption, membrane filtration, coagulation and flocculation, advanced oxidation processes, bioadsorption and activated sludge systems.^26^ However, these methods are not highly effective in removing antibiotics, and vary in efficacy from plant to plant (reviews;^23,27,28^ also, work^22^ and report^20^). Additionally, there are several studies reporting that the conditions in wastewater treatment plants favor the proliferation of antibiotic-resistant bacteria and genes that can be discharged into the environment.^29^ Wastewater and sewage sludge may be used as fertilizers, which means that they are constantly re-introduced to the food supply and natural ecosystems, despite containing antibiotic-resistant bacteria.^20,22,23,27,28^ The interaction mechanisms among humans, animals (livestock and wildlife) and the environment showcased in Fig. 1 (ref. 1) suggests the considerable challenge of implementing affordable, scalable solutions, especially in the most vulnerable, low-resource countries.

Fungi have been used as a natural, low-cost form of remediation of environmental contaminants. This is because the enzymatic system of fungi is known to remove various chemicals, through sorption or metabolism into less toxic/innocuous molecules, as observed in studies showing the degradation of hydrocarbons, polychlorinated biphenyls (PCBs)^30^ and dichlorodiphenyltrichloroethane (DDT) in water by *Phanerochaete chrysosporium*.^31^ White rot fungi were also shown to transform antibiotics in synthetic wastewater (aqueous solutions of antibiotics) or in bioreactors, although the fungi are typically in the form of cultures grown in malt-based medium,^26,32–36^ or in colonized agar.^37,38^ Data on the remediation potential of fungi grown in natural environments such as agricultural waste are limited (reviewed in ref. 39–41 ). From a practical standpoint, it would be useful and more sustainable to simply utilize fungi grown on natural waste products (as they do anyway) rather than in controlled media.

In this paper, the mycelium (the root structure) of a white rot fungus, *Ganoderma lucidum*, was grown on agricultural waste to investigate its remediation potential in synthetic wastewater. *Ganoderma lucidum* is mostly cultivated artificially because it is in high demand for its medicinal properties.^42,43^ It was chosen in this experiment because of prior published work on its use on bioremediation.^32,36,38,44,45^ Twenty antibiotics from the six classes (Amphenicols, Sulfonamides, B-lactams, Lincosamides, Quinolones, and Macrolides) were tested in this experiment, as they are commonly detected in wastewater and aquaculture farming.^13^

## 2. Materials and methods

### 2.1 Materials

LC/MS grade methanol, optima grade methanol, and toluene were obtained from Fisher Chemical . Acetonitrile was obtained from Thermo Scientific. Formic acid, HCl 37% and Na_2_EDTA were purchased from Sigma-Aldrich (St. Louis, MO). Dimethyldichlorosilane was obtained from Fisher Scientific (Hampton, NH, USA).

Antibiotic standards used in this study were from the following classes: Amphenicols (chloramphenicol (CAP), Thiamphenicol (TAP), Florfenicol (FF), Florfenicol amine (FFA)); Sulfonamides (sulfadimethoxine (SDM), Sulfasalazine (SSZ), Sulfamethoxazole (SMX), Sulfadiazine (SDZ)); B-lactams (ampicillin anhydrous (AMP), Penicillin G potassium salt (PEN-G), Penicillin V (PEN-V) and Amoxicillin (AMOX)); Lincosamides (lincomycin (LIN)); Quinolones (enrofloxacin (ENRO), Flumequine (FLU), Norfloxacin (NOR), Enoxacin (ENO)); Macrolides (erythromycin (ERYTH), Virginiamycin complex (VIRG-M1 and VIRG-S1)).

CAP (98.5%) was purchased from Crescent Chemical (Islandia, NY). TAP (99.3%), FF (98%), SDZ (99%) and AMOX (98%) were purchased from Fisher Scientific (Hampton, NH, USA). ERYTH (94.8%), ENRO (99.8%), FFA (99.3%), SDM (98.5%), AMP (99.6%), and NOR (98%) were purchased from Sigma Aldrich (St. Louis, MO). SSZ (100%), SMX (100%), LIN (98%), PEN-V (98.8%), PEN-G, FLU, ENO, and VIRG were purchased from Cayman Chemical (Ann Arbor, MI).

Isotopically labeled surrogate standards including FFA-D3 (chemical purity: 98%; isotopic purity: 98.7%), CAP-D5 (chemical purity: 98%; isotopic purity: 98.3%), LIN-D3 (chemical purity: 95%; isotopic purity: 99.6%), SMX-D4 (chemical purity: 98%; isotopic purity: 99.2%), SMZ-D4 (chemical purity: 98%; isotopic purity: 95.9%), ERYTH-D6 (chemical purity: 95%; isotopic purity: 98.1%), ENRO-D5 (chemical purity: 99.61%; isotopic purity: 99.40%), and AMP-D5 (chemical purity: 95%; isotopic purity: 99.00%), were purchased from Toronto Research Chemicals (Toronto, Ontario, Canada).

### 2.2 Biomass preparation

**Fig. 2:**
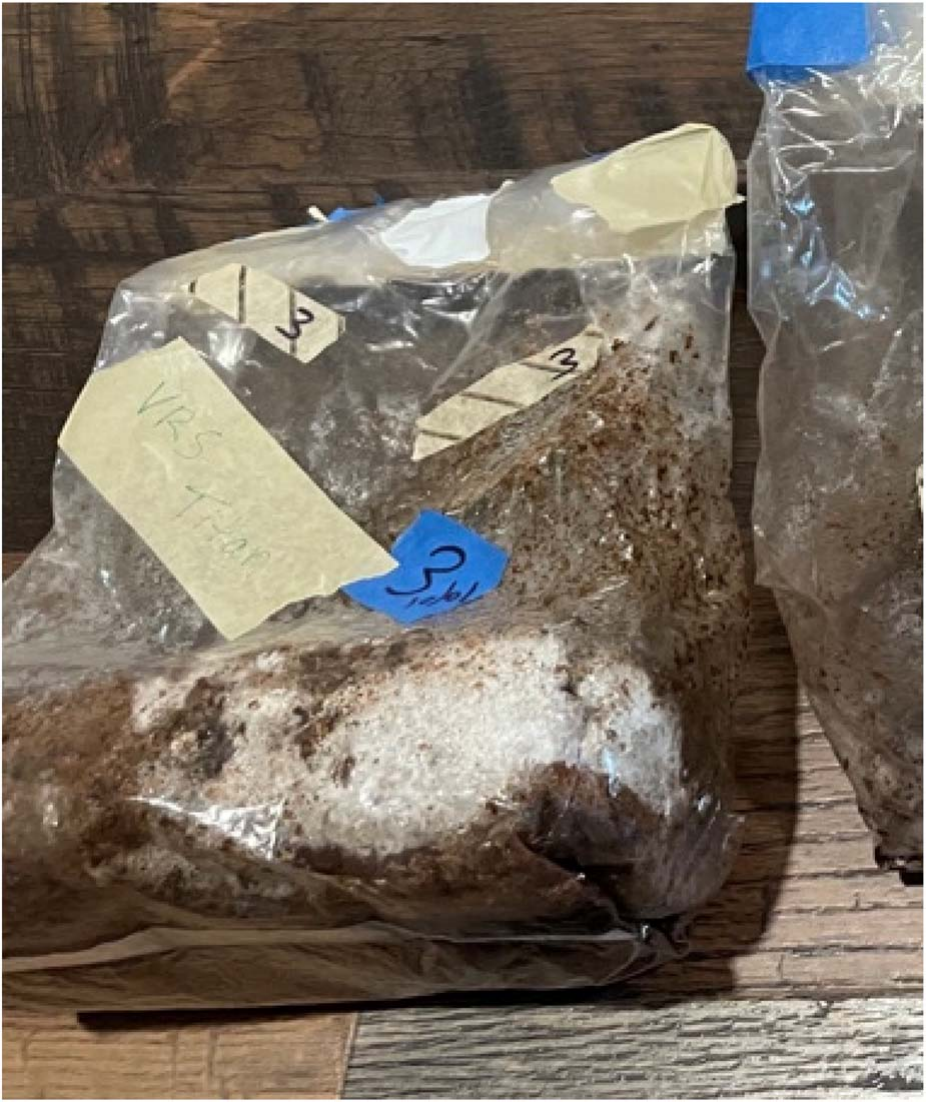
Mycelial biomass used in this study.

The biomass was prepared by mixing 140 g of oak pellets (MushroomMediaOnline, IA, USA), 98 g of almond shells from California (leftovers from a research project completed before Fall 2020) and 400 mL of filtered tap water. Used coffee grounds (3 tablespoons) from a local household and 28 g of fava stalks from a local vegetable garden (a Spring 2021 cover crop harvested, dried in the sun and then pulverized) were added as a nitrogen-rich nutrient source. The biomass mix was sterilized in a conventional autoclave and in an autoclavable bag with a filter patch (grow.bio, USA), at 121 °C and 0.103 MPa (15 psi) for 20 minutes. It was then cooled for 1 hour in a fume hood. The autoclaved biomass mix was then inoculated with 5 mL *Ganoderma lucidum* liquid culture (Root Mushroom Farm, WA, USA). After 13 days in ambient conditions (21 °C in an air-conditioned room, October 2021 in Davis, CA), sustained growth of the mycelium into the biomass was observed (Fig. 2), and the bag containing mycelium and biomass was relocated to a −80 °C freezer for storage.

### 2.3 Antibiotic mixture preparation

Antibiotics were first prepared individually in methanol (amphenicols, sulfonamides, quinolones, macrolides) or water (B-lactams). Individual stock solutions of CAP, TAP, FF, FFA, SDM, SMX, ENRO, ERYTH, VIRG, LIN, CAP-D5, SMX-D4, SMZ-D4, ERYTH-D6, ENRO-D5 and TRIM-D3 were prepared in methanol at 1 mg per mL concentration. SSZ, SDZ, and LIN-D3 were prepared in methanol at a concentration of 0.5 mg mL^−1^. ENO, NOR and FLU were prepared in methanol at concentration of 0.2 mg mL^−1^. B-lactams (AMP, PEN-G, PEN-V, AMOX and PEN-V-D5) were prepared in Milli-Q water at 1 mg mL^−1^. AMP-D5 was prepared in Milli-Q water at 0.5 mg mL^−1^. The stock solutions were diluted from 0.2–1 mg mL^−1^ to individual ‘intermediate’ solutions of 10 µg mL^−1^ using the same solvent as the stock solution.

The individual intermediate solutions of unlabeled and labeled standards were used to prepare antibiotic mixture solutions of methanol-soluble and water-soluble antibiotic standards. These mixes were prepared separately for unlabeled and labeled antibiotics. For unlabeled antibiotics, both methanol-soluble and water-soluble mixes were prepared at a concentration of 400 ng mL^−1^. For labeled antibiotics, both methanol-soluble and water-soluble mixes were prepared at a concentration of 1000 ng mL^−1^. The water-soluble and methanol-soluble antibiotics were mixed at a 1 : 1 ratio before use. The unlabeled and labeled standards were mixed separately, as they were needed for different purposes: the unlabeled mixes were used for spiking water samples, and the labeled mixes were spiked to each sample right before extraction, for the purpose of quantification.

For methanol-soluble unlabeled antibiotic standards (*n* = 15; Table A.1 SI), 30 µL of each antibiotic from their individual intermediate solutions (10 µg mL^−1^) were added to a 2 mL amber glass LC vial. The mixture was evaporated under nitrogen and reconstituted in 750 µL LC-MS methanol. For water-soluble antibiotic standards (*n* = 4; Table A.1 in the SI), 630 µL Milli-Q water was added to a 2 mL amber glass LC vial followed by adding 30 µL of each of the four unlabeled water-soluble antibiotic standards (from their individual intermediate solutions (10 µg mL^−1^)).

For methanol-soluble labeled antibiotic standards, 50 µL of each labeled standard (from their individual intermediate solutions (10 µg mL^−1^)) were added to a 2 mL amber glass LC vial. Samples were vortexed, dried under nitrogen, and reconstituted in 500 µL LC-MS methanol. For water-soluble labeled antibiotic standards, 400 µL Milli-Q water was added to a 2 mL amber glass LC vial followed by 50 µL of the two water soluble standards from their individual intermediate solutions (10 µg mL^−1^). Water-soluble and methanol-soluble antibiotic mixes were mixed at a 1 : 1 ratio before the experiment, resulting in working mix of unlabeled antibiotic standards at concentration of 200 ng mL^−1^ and working labeled standard mix at concentration of 500 ng mL^−1^.

### 2.4 Glass silanization prior to antibiotic treatment

Some antibiotics including quinolones were shown to adsorb onto the glass surface, likely due to interactions with the glass silanol groups.^46,47^ Glassware was therefore silanized using dimethyldichlorosilane (DMDCS) prior to the experiment, in order to cover the silanol groups on the glass surface, using the method described in ref. 48. Briefly, clean and dry Pyrex glass vials (50 mL) were pre-washed with detergent-free soap and water, and rinsed 5 times with distilled water. The vials were treated with 5 mL 5% DMDCS in toluene solution. Silanization was completed by rinsing and vortexing three more times with 5 mL of 5% DMDCS in toluene, 5 mL toluene, 5 mL methanol, and 5 mL Milli-Q water, respectively. Vials were left overnight to dry.

### 2.5 Experimental design and mycelium treatment

Mycelial biomass (*Ganoderma lucidum* grown on the substrate described in Section 2.2) was thawed on ice for approximately 90 minutes. Approximately, 1 g of biomass was weighed and placed into 50 mL silanized Pyrex glass tubes. Milli-Q water (50 mL) was then added. Mycelium–water samples were spiked with 20 ng of each unlabeled antibiotic per 25 mL water (total of 20 antibiotics belonging to six different classes; Table A.1, SI) and incubated for 0 (baseline) or 3 days (*n* = 4 samples per incubation period). A parallel set of samples (*n* = 4 samples per timepoint) contained 20 ng antibiotics in 25 mL Milli-Q water without mycelium, as a negative control. Each sample was set in its own independent 50 mL of silanized Pyrex tube. Samples corresponding to Day 3 were covered in foil and shaken at room temperature using an Excella E24 shaker at 100 rpm for 3 days. On Day 3, the Day 0 (baseline) samples were completed in separate Pyrex tubes. Additionally, one water method blank containing 25 mL of Milli-Q water and no antibiotics (*n* = 1 on Day 0 and Day 3), as well as one matrix blank containing mycelium and water only (no antibiotics; *n* = 1 on Day 0 and *n* = 1 on Day 3) were incorporated into the design to control for any background antibiotics potentially coming from the water itself or the mycelium, respectively. Antibiotics were extracted from both Day 0 and Day 3 samples as described below.

The final design and sample size for each incubation day (*i.e.* Day 0 and Day 3) were as follows:

*n* = 1, Water method blank (water only) = 25 mL Milli-Q water.

*n* = 1, Matrix method blank (water + mycelium) = 0.5 g mycelial biomass in 25 mL Milli-Q water.

*n* = 4, Control sample (water + antibiotics), named “Control” in the statistical analysis = 20 ng antibiotics in 25 mL Milli-Q water.

*n* = 4, Treatment sample (water + mycelial biomass + antibiotics), named “Treated” in the statistical analysis = 0.5 g mycelial biomass + 20 ng antibiotics in 25 mL Milli-Q water.

After incubation, the samples were centrifuged using SORVALL RT 6000D at 3000 rpm for 10 minutes at room temperature. One water–mycelial biomass Day 3 sample and one water–mycelial biomass antibiotic Day 3 sample broke in the centrifuge. To prevent the rest of the tubes from breaking, including all tubes from Day 0, samples were vortexed and allowed to sit for a few minutes. Water samples were then transferred to 40 mL non-silanized glass vials for the extraction of antibiotics.

### 2.6 Antibiotics extraction

The water extracts (in non-silanized glass vials) were spiked with 20 ng of surrogate standard mixture containing CAP-D5, FFA-D3, TRIM-D3, SMZ-D4, SMX-D4, LIN-D3, ENRO-D5, ERYTH-D6, AMP-D5, and PEN-V-D5 (500 ng per mL per surrogate standard). Then, 1340 µL of 0.05 M Na_2_EDTA in water (corresponding to 0.1% Na_2_EDTA) was added to the glass vials. Aliquots were let to stay for 1 hour covered with aluminum foil with occasional shaking before solid phase extraction (SPE).

### 2.7 Solid phase extraction (SPE)

Antibiotics were extracted from the water samples using Waters OASIS HLB cartridges (60 mg, 3 cm). The cartridges were pre-conditioned with methanol (5 mL), Milli-Q water (5 mL), and pH = 2.5 water (5 mL) made by adding 90 µL HCL (37%) to 150 mL Milli-Q water in a flask and verifying the pH with litmus paper. The water sample extracts were loaded onto the conditioned HLB cartridges and allowed to elute. The SPE cartridges were then washed with 6 mL Milli-Q water and the SPE cartridges were dried under the vacuum manifold (Supelco Visiprep 24 SPE) for five minutes at 0.117 MPa (17 psi). Antibiotics were eluted into 8 mL glass vials using 5 mL Optima grade methanol. The vials were stored overnight in a −20 °C freezer. The following day, the samples were evaporated under nitrogen for approximately two hours. Samples were reconstituted in 1 mL LC-MS methanol : water (1 : 1), vortexed for 3 minutes, transferred to 2 mL centrifuge tubes, and centrifuged for 2 min at 12000 rpm at 0 °C (Eppendorf, 5424 R, 13 523×*g*). The samples were then transferred into filter centrifuge tubes (two tubes per sample each containing approx. <500 µL of sample per tube). The actual sample amount pipetted was 480 µL per tube. The filter tubes were centrifuged for 10 minutes at 12000 rpm (Eppendorf, 5424 R, 13 523×*g*). The filters were discarded, and the extract was transferred to LC vials with slit caps for UPLC-MS/MS analysis (total volume of 960 µL in LCMS vial).

### 2.8 LC-MS/MS instrumentation

Antibiotic analysis was performed on an Agilent ultra-high pressure liquid chromatography system coupled to a 6460 Agilent triple quadrupole (LC-MS/MS). Chromatographic separation of the antibiotics mixture was performed on an AQUITY BEH C18 column (100 × 2.1 mm, 1.8 µm), using 0.1% formic acid in water (mobile phase A) and 0.1% formic acid in acetonitrile (mobile phase B) running at a flow rate of 0.300 mL min^−1^ and column temperature of 30 °C. MS/MS analysis was performed using Agilent Jetstream electrospray ionization (ESI) operating on both positive and negative mode. MS source parameters were as follows: sheath gas temperature of 375 °C, sheath gas flow of 8 L min^−1^, drying gas temperature of 250 °C, nozzle voltage of 0 V, nebulizer gas pressure of 40 psi and capillary voltage of 3500 V. Collision-induced dissociation was carried out using nitrogen at the collision cell. A total of ∼140 samples were run on the LC-MS/MS, including samples, water controls, blanks and calibration curves.

### 2.9 Antibiotics quantification

Antibiotic concentrations in water samples were calculated by the internal standard calibration method where isotopically labeled surrogates were used to correct for recoveries and unlabeled standards were used to correct for the detector response factor. An 11-point standard calibration curve (0.01–100 ng mL^−1^) containing a fixed amount of surrogate standard (20 ng mL^−1^) was made to derive the response factor. Calibration curves were generated by quadratic regression and 
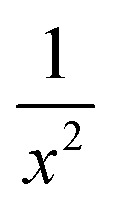
 weighting factor was applied. Peaks were integrated and analyzed using MassHunter Workstation Software, QQQ Quantitative Analysis (Agilent Technologies, Inc.).

### 2.10 Statistical analysis

MATLAB (Mathworks) was used to perform a *χ*^2^ goodness-of-fit tests on the data and its log 10 transformed value. The rest of the statistical analysis was conducted with Graphpad Prism9 (GraphPad) using for most cases a two-way repeated ANOVA (time as repeated measure and treatment as a main factor) with a 5% significance level applied to the log 10 values (for reduced data skewness). For 3 out of the 20 sets of log 10 data (namely, SSZ, SDM and SDZ), the residuals were found not to be normally distributed (a key requirement for ANOVA). Therefore, a mixed-effects model (with Šidák's multiple comparison) was employed in place of ANOVA, also with Prism9 software.

## 3. Results

**Fig. 3:**
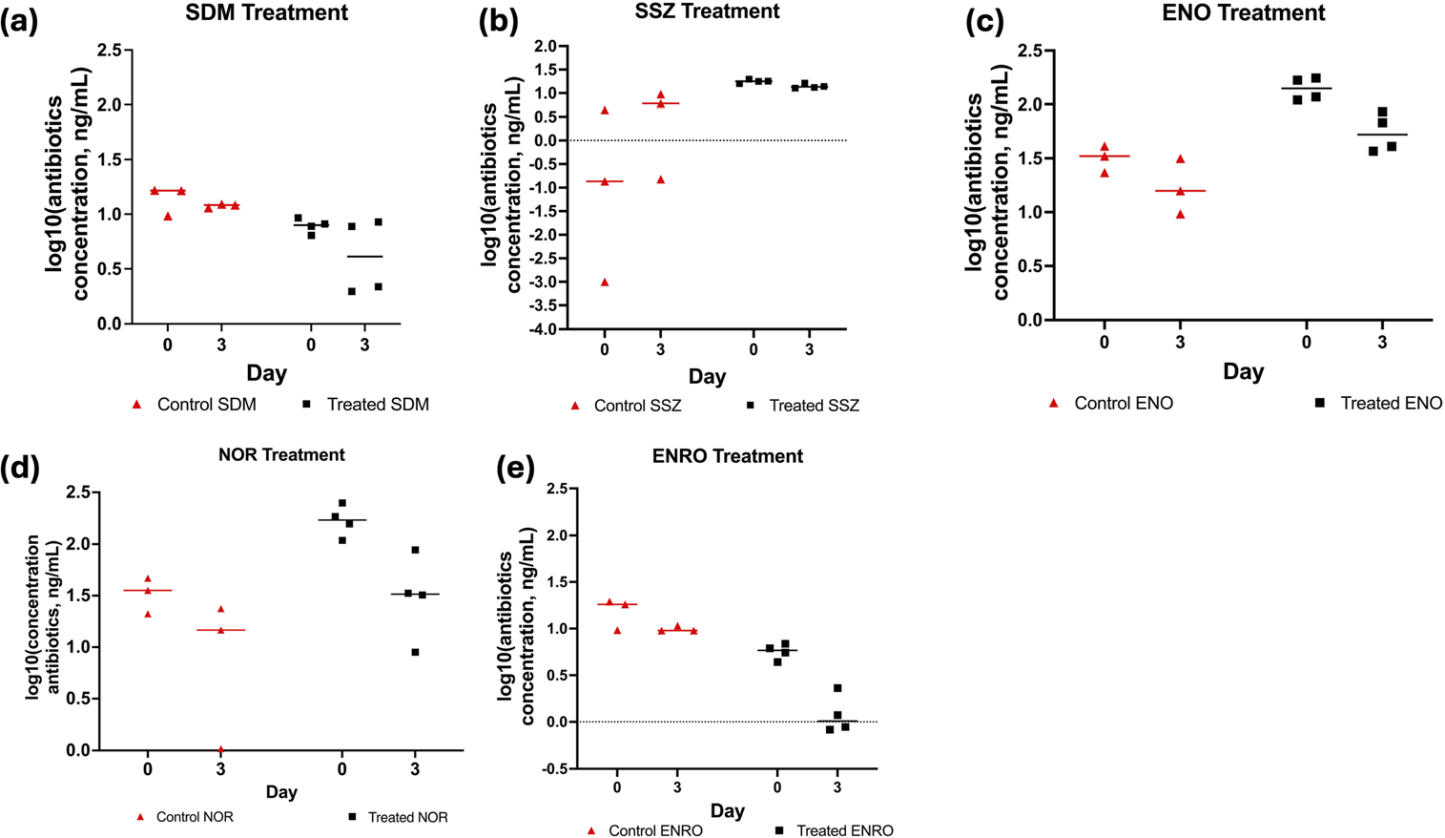
(a) Plot of log 10 (concentrations [ng mL^−1^]) of control and treated solutions with sulfonamide SDM. The solid line is the median of the data points for that group. (b) Plot of log 10 (concentrations [ng mL^−1^]) of control and treated solutions with sulfonamide SSZ. The solid line is the median of the data points for that group. (c) Plot of log 10 (concentrations [ng mL^−1^]) of control and treated solutions with quinolone ENO. The solid line is the median of the data points for that group. (d) Plot of log 10 (concentrations [ng mL^−1^]) of control and treated solutions with quinolone ENRO. The solid line is the median of the data points for that group. (e) Plot of log 10 (concentrations [ng mL^−1^]) of control and treated solutions with quinolone ENRO. The solid line is the median of the data points for that group.

As shown in Fig. 3a–e and Tables A.6–A.8, Fig. A.3 in the SI, there was a significant time effect for SSZ and PEN-G antibiotics on Day 3 compared to Day 0 (baseline). There were significant treatment and time effects for ERYTH and VIRG-S1 antibiotics, with the control samples exhibiting a larger decrease in concentration over time compared to antibiotic-spiked samples (Fig. A.6 in the SI). For SDM, ENO, ENRO, NOR antibiotics, the mycelium + biomass treatment reduced antibiotic concentrations more than the control samples on Day 3, compared to Day 0 (Fig. 3). No significant reductions were seen in the parallel samples of water containing the antibiotics but lacking the mycelium after 3 days. For SSZ, the control group appeared to increase after 3 days, likely due to sorption/desorption with the glassware; this increase was not seen in the mycelium + biomass treated group possibly due to the biomass removing it from the system upon desorption from the glass. Overall, as seen in Fig. 3, the mycelium + biomass treatment appeared to be most useful in remediating quinolones (particularly ENRO), a notoriously recalcitrant class of antibiotics,^49^ followed by the sulfonamide, SDM. The analysis of matrix effects (ME) from the water–mycelium matrix following standard procedures^46,50^ is reported in the SI; as shown, matrix effects were comparable between the groups.

## 4. Discussion

The main finding of this study is that a 3-day treatment of antibiotic-containing water with mycelium grown on agricultural waste reduced antibiotic concentrations in water by up to 82.4%. Our findings provide proof-of-concept demonstration of the use of naturally grown fungus for antibiotic remediation in contaminated water.

The effects of mycelium incubation on antibiotic reduction in water were mostly greater than previously reported with other fungal strains or similar strains grown on different media, highlighting the importance of utilizing the right substrate (*i.e.* agricultural waste) for maximizing fungal growth and potency. With respect to published results in mycelium cultures without agricultural waste, Vasiliadou *et al.*^36^ reported a less than 20% change after an exposure of 7 days to sulfamethoxazole (SMX) by *Ganoderma lucidum* grown with malt extract. Chakraborty and Abraham^44^ reported 100% removal after 4 days of exposure to enrofloxacin (ENRO) by *Ganoderma lucidum*, findings which are comparable to our results (−82.4% after 3 days of treatment). Martens *et al.*^51^ showed considerably less success when enrofloxacin (ENRO) was degraded by other mycelial species (varying between 0.19% for white rot fungus, and 25.6% for brown rot fungus, and after 56 days), likely due to differences in the species used to degrade antibiotics. Čvančarová *et al.*^35^ showed that norfloxacin (NOR) degraded by up to 100% with *Irpex lacteus* after 10 days, and by 10% with *Dichomitus squalens* after 10 days. With all these studies, a longer duration was needed to achieve similar antibiotic reduction magnitude as in this study, where it was achieved within 3 days.

The reduction in antibiotic concentrations after 3-day mycelium incubation could be due to increased adsorption of antibiotics to the mycelium surface, or internalization and degradation by mycelium enzymes. Adsorption is an unlikely mechanism as it is non-specific, meaning that all 20 antibiotics applied would have adhered to the mycelium non-selectively. Enzymatic degradation is more plausible, given that 4 antibiotics belonging to the quinolone and sulfonamide families decreased in concentration after 3 days. However, without enzyme assays, adsorption cannot be completely ruled out. Studies have demonstrated the ability of fungi to oxidize antibiotics *in vitro via* peroxidases, ligninase, xylanases and cellulases.^45^

There are several limitations worth noting. Since our exposure lasted only 3 days, we do not know whether the results would be further enhanced with prolonged incubation with the mycelium. Additionally, different agri-waste substrates may have different priming effects on mycelium potency (in terms of antibiotic reduction capability) and growth potential. Recent work co-authored by La Saponara^52^ highlighted the importance of the substrate's chemistry (assessed in terms of carbon : nitrogen ratios) for the evolution (colonization, mechanical strength and competition with pathogens) of the chosen mycelium species.^52^ Studies involving prolonged incubation of mycelium grown on different agri-waste substrates (*e.g.* grape or tomato pomace, or any locally available agri-waste) with contaminated water, are needed to optimize the remediation potential of mycelium, building upon substrate chemistry to guide the process. A final limitation relates to the risk of biomass contamination with antibiotic-degrading bacteria. Although the biomass was sterilized before inoculation, it would have been useful to test a separate control group consisting of water-uninoculated biomass plus antibiotics to test for symbiotic effects involving bacteria.

## 5. Conclusions

In our exploratory investigation, we showed that a mycelium (*Ganoderma lucidum*) grown on agricultural waste reduces the concentration of selected antibiotics in synthetic wastewater. In just 3 days, the concentrations of some quinolone and sulfonamide antibiotics were reduced compared to baseline, and with respect to spiked synthetic water without mycelium/biomass (as shown through statistically significant reduction corroborated by tolerable matrix effects). The results on the quinolone antibiotics are particularly promising because of their extensive consumption worldwide (*e.g.* ref. 13 and 53), and the inability of conventional wastewater treatment plants to remove them, thus fostering antibiotic resistance and harming the environment.^54^ In summary, this first proof-of-concept work indicates that mycelium grown on re-purposed agricultural waste is a promising, novel method to remove from water certain antibiotics. Additional studies are needed to understand whether removal mechanisms by the mycelium are due to enzymatic degradation or surface interactions with antibiotics..

## Author contributions

All authors whose names appear on the submission (1) made substantial contributions to the conception or design of the work: V. J., V. L. S., S. E. and A. Y. T designed the experiments. V. J. and S. E. performed the experiments and analyzed the data. V. J. wrote the original draft. V. J. and S. E. edited the manuscript. V. L. S. and A. Y. T. reviewed and edited the manuscript and carried out the statistical analysis. V. L. S. and A. Y. T. share equally the intellectual property of this work and are co-corresponding authors. (2) Approved the version to be published, and (3) agree to be accountable for all aspects of the work in ensuring that questions related to the accuracy or integrity of any part of the work are appropriately investigated and resolved.

## Conflicts of interest

The authors declare that they have no relevant financial or non-financial interests to disclose.

## Abbreviations

AMOX: Amoxicillin
AMP: Ampicillin
CAP: Chloramphenicol
ENRO: Enrofloxacin
ENO: Enoxacin
ERYTH: Erythromycin
FF: Florfenicol
FFA: Florfenicol amine
FLU: Flumequine
LIN: Lincomycin
NOR: Norfloxacin
PEN-G: Penicillin G
PEN-V: Penicillin V
SPE: Solid phase extraction
SDM: Sulfadimethoxine
SDZ: Sulfadiazine
SMX: Sulfamethoxazole
SSZ: Sulfasalazine
TAP: Thiamphenicol
VIRG-M1: Tilmicosin: Virginiamycin M1
VIRG-S1: Virginiamycin S1

## Supplementary Material

RA-016-D5RA06482A-s001

## Data Availability

The raw data for this study are reported throughout the manuscript in the form of scatter plots. Raw data can also be made available upon request from the authors. Supplementary information (SI) is available. See DOI: https://doi.org/10.1039/d5ra06482a.
