# Supplementary material for: Use of Ganoderma lucidum grown on agricultural waste to remove antibiotics from water
Source: RSC Adv. 2026 Jan 5;16(2):1499–508. doi: 10.1039/d5ra06482a (PMC12767782; doi:10.1039/d5ra06482a)
Supplement: RA-016-D5RA06482A-s001 [file RA-016-D5RA06482A-s001.pdf]

This journal is © The Royal Society of Chemistry 2025

Appendix/Supplementary Information

Use of *Ganoderma lucidum* Grown on Agricultural Waste to Remove Antibiotics from Water

Vanessa Juarez[1,2], Shiva Emami [2], Ameer Y. Taha [2], Valeria La Saponara [1]
ataha@ucdavis.edu , vlasaponara@ucdavis.edu

[1] Department of Mechanical and Aerospace Engineering, University of California, Davis, One Shields Avenue, Davis, CA, USA 95616

[2] Department of Food Science and Technology, University of California, Davis, One Shields Avenue, Davis, CA, USA 95616

A.1 Antibiotic Standard and Other Information

This information is provided in **Tables A.1-A.4**.

Table A.1: Antibiotic Standards[46]

Class	Antibiotic Abbreviation	Antibiotic Name	Catalogue Number
Methanol Soluble:			
**Amphenicols**	CAP	Chloramphenicol	40524
	FF	Florfenicol	73231-34-2
	FFA	Florfenicol amine	F405773
	TAP	Thiamphenicol	AAJ63575-03
**Sulfonamides**	SDM	Sulfadimethoxine	122-11-2
	SDZ	Sulfadiazine	68-35-9
	SMX	Sulfamethoxazole	23613
	SSZ	Sulfasalazine	15025
**Lincosamides**	LIN	Lincomycin	21526
**Quinolones**	ENO	Enoxacin	16956
	ENRO	Enrofloxacin	33699
	FLU	Flumequine	21645
	NOR	Norfloxacin	70458-96-7
**Macrolides**	ERYTH	Erythromycin	114-07-8
	VIRG-M1 + VIRG-S1	Virginiamycin M1 + Virginiamycin S1	14503

Water Soluble:			
**B-lactams**	AMOX	Amoxicillin	26787-78-0
	AMP	Ampicillin	69-53-4
	PEN-G	Penicillin G	21615
	PEN-V	Penicillin V	23635

Table A.2: Surrogate Standards[46]

Surrogate Abbreviation	Surrogate Name	Catalogue Number
	Methanol Soluble:	
**CAP – D5**	*Chloramphenicol – D5*	C325033
**FFA – D3**	*Florfenicol amine – D3*	F405773
**TRIM - D3**	*Trimethoprim - D3*	T79618
**SMZ - D4**	*Sulfamethazine - D4*	S699072
**SMX – D4**	*Sulfamethoxazole – D4*	S699087
**LIN – D3**	*Lincomycin – D3*	L466202
**ENRO – D5**	*Enrofloxacin – D5*	E557802
**ERYTH – D6**	*Erythromycin – D6*	E649953
	Water Soluble:	
**AMP – D5**	*Ampicillin – D5*	A634337
**PEN-V – D5**	*Penicillin V – D5*	*26786*

Table A.3: Unlabeled Standard & Unlabeled Surrogate Mix Concentrations[46]

Calibration Level	Methanol:Water (1:1)	Unlabeled - Standard Mix	Labeled - Surrogate Mix
0.001 ng/mL	1919 µL	250 µL	80 µL
0.01 ng/mL	1910 µL	100 µL	80 µL
0.1 ng/mL	1820 µL	10 µL	80 µL
0.25 ng/mL	1670 µL	1 µL	80 µL
0.5 ng/mL	1915 µL	5 µL	80 µL

1 ng/mL	1910 µL	10 µL	80 µL
2 ng/mL	950 µL	10 µL	40 µL
5 ng/mL	374 µL	10 µL	16 µL
10 ng/mL	364 µL	20 µL	16 µL
20 ng/mL	344 µL	40 µL	16 µL
50 ng/mL	284 µL	100 µL	16 µL
100 ng/mL	184 µL	200 µL	16 µL

Table A.4: Mobile phases[46]
A: 0.1 % formic acid in water
B: 0.1 % formic acid in Acetonitrile

	Time	A	B	Flow	Pressure
1	8.00 min	80.00 %	20.00 %	0.300 mL/min	600.00 bar
2	11.00 min	60.00 %	40.00 %	0.300 mL/min	600.00 bar
3	13.00 min	0.00 %	100.00 %	0.300 mL/min	600.00 bar
4	15.00 min	0.00 %	100.00 %	0.300 mL/min	600.00 bar
5	17.00 min	90.00 %	10.00 %	0.300 mL/min	600.00 bar
6	20.00 min	90.00 %	10.00 %	0.300 mL/min	600.00 bar

A.2 Matrix Effects

Matrix effects (ME) were calculated as the ratio of the peak area of the antibiotic standards (unlabeled and labeled) in sample spiked after extraction (ME-D0) to the peak area of antibiotic standards in pure solvent (**Table A.5**). Concentrations for quinolones including NOR and ENO in Day 0 samples (n=4) were higher than the expected concentration of 20 ng/ml (Figure 2). The mean concentration of NOR in Water-Mycelium-Antibiotic (treated) samples (n=4) was 198.59

ng/mL ± 51.11 ng/mL SD. The mean concentration of ENO was 161.35 ng/mL ± 48.49 ng/mL SD. In assessing matrix effects for the three significant quinolones (enrofloxacin ENRO, norfloxacin NOR, and enoxacin ENO), the surrogate ENRO-D5 (enrofloxacin-D5) was used. Analytes NOR and ENO show different matrix effects compared to the surrogate ENRO-D5. Particularly, ENO showed ion enhancements (87% and 108%), NOR did not show major ion suppression or ion enhancement (126% and 181%), and ENRO-D5 showed ion suppression (42% and 21%). The discrepancies in the matrix effects of the analytes NOR and ENO (quinolones) and surrogate ENRO-D5 may affect the accuracy in concentrations, resulting in overestimated concentrations.

ENRO did not exhibit high Day 0 concentrations as the other two quinolones. This coincides with ENRO and ENRO-D5 behaving similarly in terms of matrix effects (**Table A.6**). Low concentrations found for ENRO (6.14 ng/ml ± 0.62 ng/mL SD) may be due to quick adsorption to biomass on Day 0. Future studies are required to test whether the biomass is adsorbing the antibiotics and therefore reducing the initial concentration levels.

Table A.5: Matrix effects for 20 antibiotic standards (unlabeled standard) and 10 isotopically labeled standards in Day 0. Matrix effects were calculated by dividing the peak area (response) in Water-Mycelium samples (WM) spiked with standards right after extraction (ME, n=2) to the peak area in pure solvent.

Antibiotic Abbreviation	Antibiotic Name	Matrix Effects: Day0 Sample 1	Matrix Effects: Day0 Sample 2
**CAP-D5**		**99%**	**16%**
CAP	*Chloramphenicol*	121%	46%
TAP	*Thiamphenicol*	31%	7%
FF	*Florfenicol*	64%	20%
**FFA-D3**		**59%**	**30%**
FFA	*Florfenicol amine*	43%	26%
**SMX-D4**		**142%**	**45%**
SMX	*Sulfamethoxazole*	95%	49%
SDM	*Sulfadimethoxine*	87%	49%
VIRG-M1	*Virginiamycin M1*	90%	64%

VIRG-S1	*Virginiamycin S1*	158%	168%
**SMZ-D4**		**81%**	**22%**
SSZ	*Sulfasalazine*	66%	47%
SDZ	*Sulfadiazine*	50%	25%
**AMP-D5**		**116%**	**73%**
AMP	*Ampicillin*	79%	55%
AMOX	*Amoxicillin*	83%	61%
**PEN-V-D5**		**84%**	**37%**
PEN-G	*Penicillin G*	136%	77%
PEN-V	*Penicillin V*	108%	79%
**LIN-D3**		**132%**	**54%**
LIN	*Lincomycin*	105%	69%
**ENRO-D5**		**42%**	**21%**
ENRO	*Enrofloxacin*	48%	48%
NOR	*Norfloxacin*	126%	181%
ENO	*Enoxacin*	87%	108%
**TRIM-D3**		**97%**	**36%**
FLU	*Flumequine*	104%	80%
**ERYTH-D6**		**65%**	**13%**
ERYTH	*Erythromycin*	50%	16%

It should be noted that the matrix effects results (ME 1 and ME 2, **Table A.5**) showed high variation. This is likely because thee samples were only vortexed and not centrifuged. This could likely result in inconsistent extraction of matrix components into the supernatant phase, and thereby variable matrix effects. Matrix effects show that the ionization of the antibiotic analytes is affected by matrix compounds within the sample extracts due to lack of silanization of Day 0 samples. Stability is known to be affected by temperature, solvent composition and, in this case, container type[47]. However, there were good extraction recoveries observed for the majority of compounds. Lower recoveries could be caused by the presence of organic matter in the matrix, with surfactant properties that could increase signal intensity, and cause suppression by promoting ionization in the positive electrospray (Yang and Carlson, J Chromatogr A. 2004; 1038:141). Higher recoveries are found to possibly be due to more antibiotic adsorption into the biomass than the mycelium.

A.2 Additional Information on Statistical Analysis, Additional Plots

The results of the statistical analyses are reported in **Tables A.6-A.8**:

Table A.6: Results of statistical analysis on log10(antibiotics concentrations), [ng/mL])

Class	Name	Significant (p<0.05)?	(If significant) reason for change/ control or treated ?
**Amphenicols**	CAP	Yes	Treatment/same
	FF	No	

	FFA	No	
	TAP	Yes	Treatment/same
**Sulfonamides**	SDM	Yes	Treatment/**treated**
	SDZ	No	
	SMX	No	
	SSZ	Yes	Time, interaction/**treated**
**Lincosamides**	LIN	No	
**Quinolones**	ENO	Yes	Time, treatment/**treated**
	ENRO	Yes	Time, treatment, interaction/**treated**
	FLU	Yes	Treatment/same
	NOR	Yes	Time, treatment/**treated**
**Macrolides**	ERYTH	Yes	Time, treatment, interaction/control
	VIRG-M1	Yes	Treatment/same
	VIRG-S1	Yes	Time, treatment/control
**B-lactams**	AMOX	Yes	Treatment/same
	AMP	Yes	Treatment/same
	PEN-G	Yes	Time/control
	PEN-V	No	

Table A.7: Results of effective treatment on log10(concentrations [ng/mL]) of quinolones ENO, ENRO, NOR, at 5% significance level, with two-way repeated ANOVA

**Source of Variation for ENO**	**% of total variation**	**P value**	**Significant?**
Interaction Time x ENO Treatment	0.8524	0.48	No
Time	21.32	0.0124	Yes
ENO Treatment	60.7	0.0017	Yes
Variation among Subjects	8.096	0.4576	No
**Source of Variation for ENRO**	**% of total variation**	**P value**	**Significant?**
Interaction Time x ENRO Treatment	7.763	0.0106	Yes
Time	23.23	0.001	Yes
ENRO Treatment	56.95	0.0007	Yes
Variation among Subjects	5.034	0.2255	No
**Source of Variation for NOR**	**% of total variation**	**P value**	**Significant?**
Interaction Time x NOR Treatment	0.1139	0.8723	No
Time	33.88	0.0331	Yes
NOR Treatment	30.76	0.0213	Yes
Variation among Subjects	14.07	0.6436	No

Table A.8: Results of effective treatments effective treatment on log10(concentrations [ng/mL]) on sulfonamides SDM, SSZ, at 5% significance level, with mixed-effects analysis

**Mixed-effects model for SDM**		
Fixed effects (type III), SDM	**P value**	**Significant?**
Time	0.125	No
SDM Treatment	0.0329	Yes
Interaction Time x SDM Treatment	0.2898	No
Random effects, SDM	**Stand Dev**	**Variance**
Subject variations	0.1032	0.01064
Residual	0.1723	0.02967
**Was the matching effective (SDM)?**		
Chi-square, df	0.3613, 1	
P value	0.5478	
Is there significant matching (P<0.05)?	No	
**Mixed-effects model for SSZ**		
Fixed effects (type III), SSZ	**P value**	**Significant?**
Time	0.0393	Yes
SSZ Treatment	0.0657	No
Interaction Time x SSZ Treatment	0.0233	Yes
Random effects, SSZ	**Stand Dev**	**Variance**
Subject variations	0.8266	0.6833
Residual	0.4281	0.1833
**Was the matching effective (SSZ)?**		
Chi-square, df	4.861, 1	
P value	0.0285	
Is there significant matching (P<0.05)?	Yes	

Plots of the log10 concentrations (in ng/mL) of 15 antibiotics are shown in **Figures A.1-A.6.**

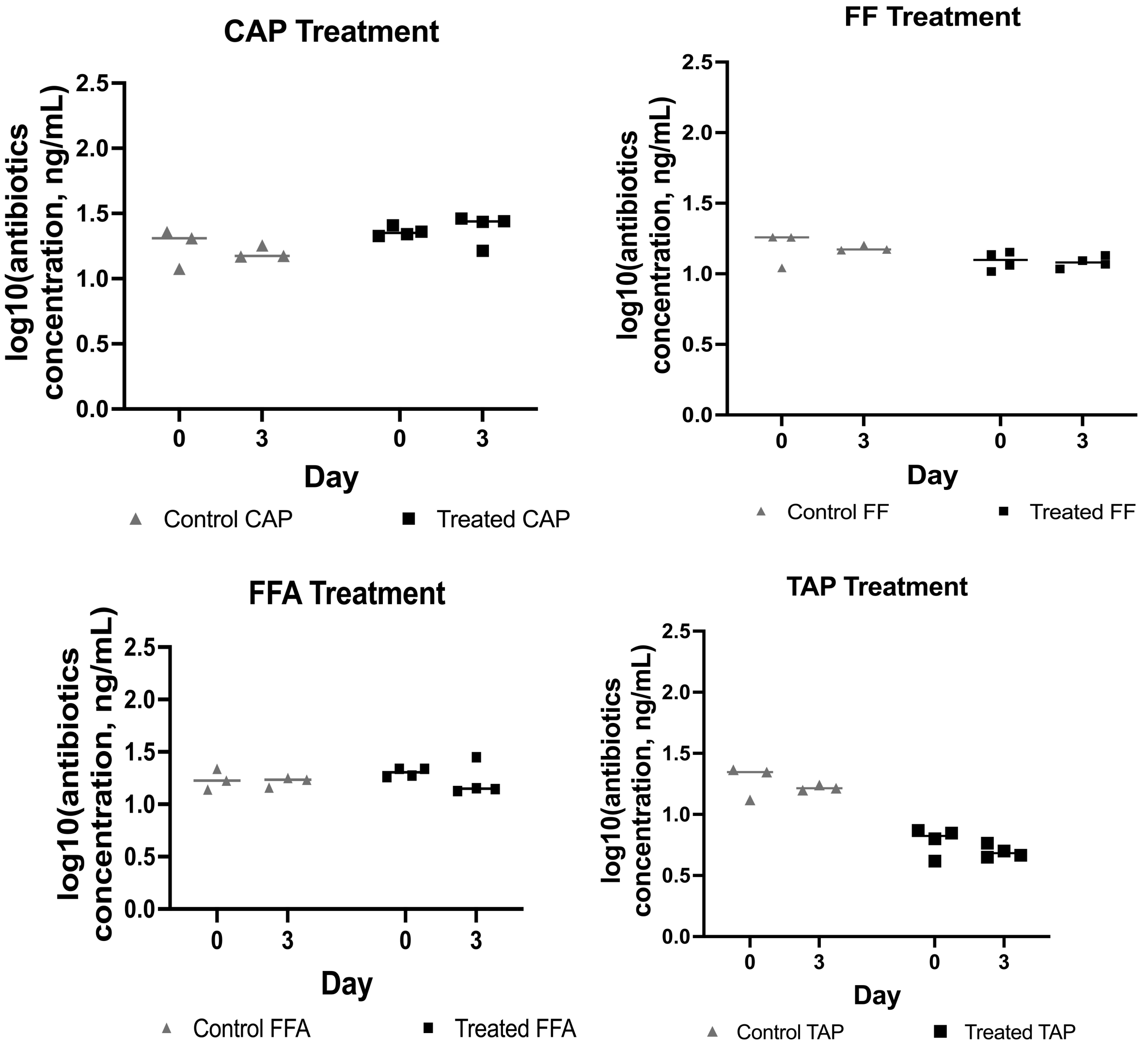

Figure A.1: log10(concentration) changes for amphenicols CAP, FF, FFA and TAP. The solid line is the median of the data points for that group.

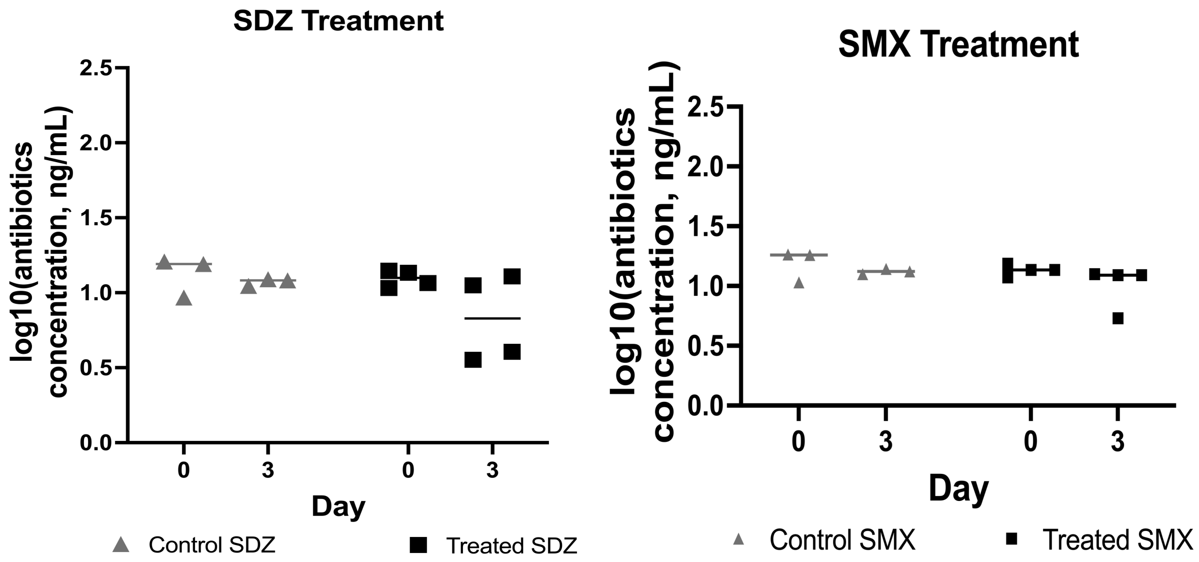

Figure A.2: log10(concentration) changes for sulfonamides SDZ and SMX. The solid line is the median of the data points for that group.

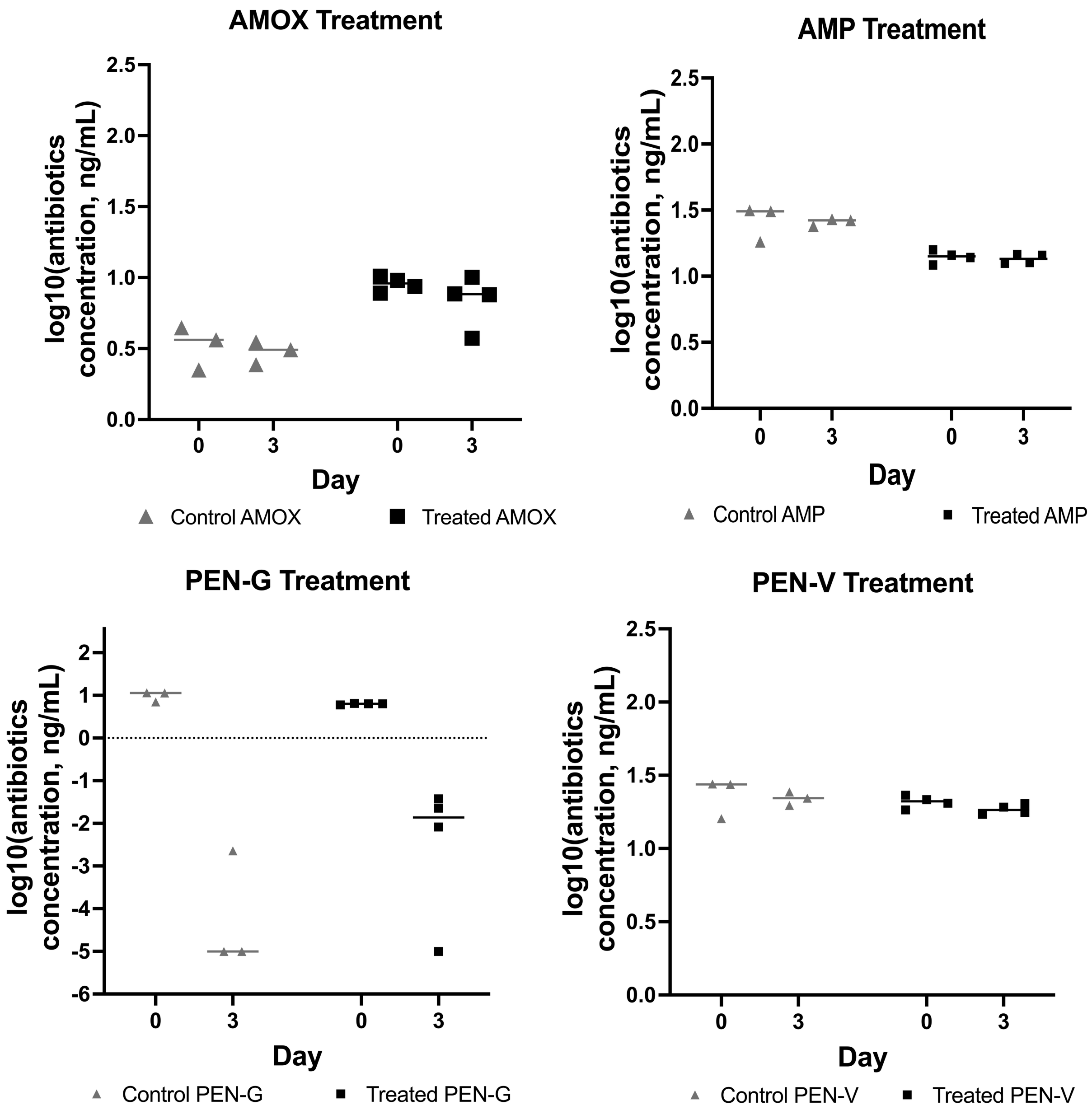

Figure A.3: log10(concentration) changes for B-lactams AMOX, AMP, PEN-G, PEN-V. The solid line is the median of the data points for that group.

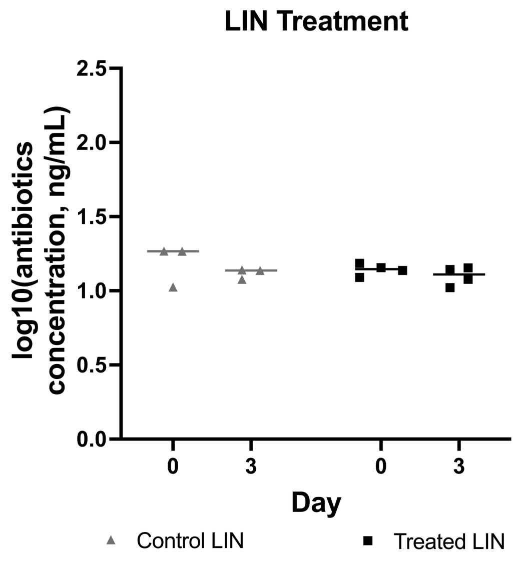

Figure A.4: log10(concentration) changes for lincosamide LIN. The solid line is the median of the data points for that group.

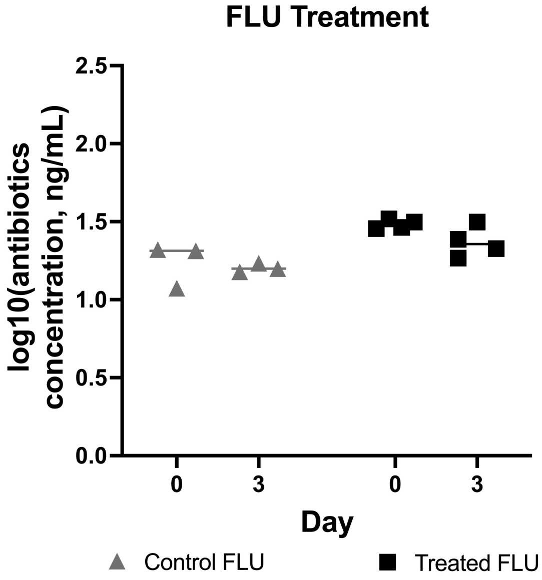

Figure A.5: log10(concentration) changes for quinolone FLU. The solid line is the median of the data points for that group.

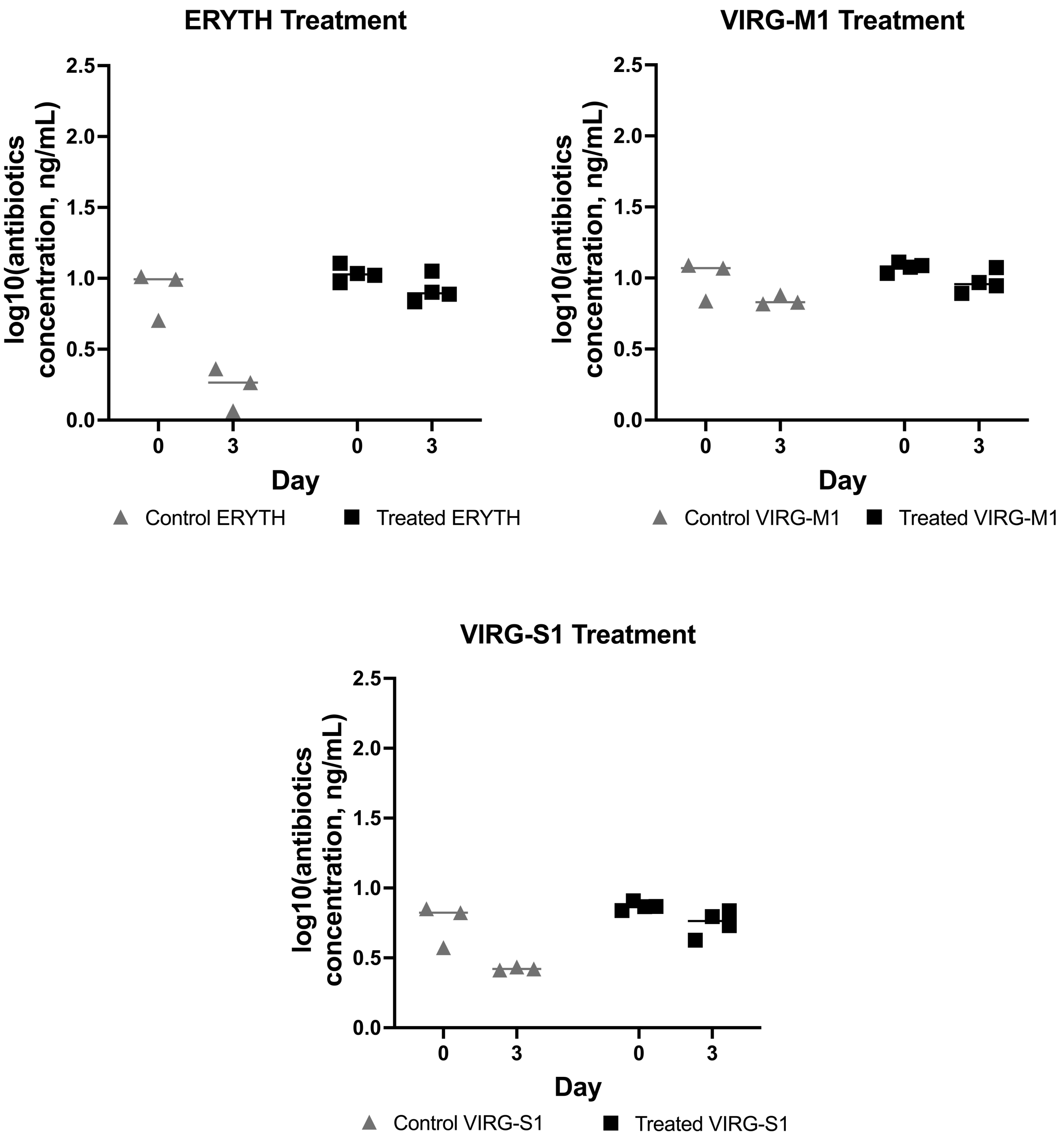

Figure A.6: log10(concentration) changes for macrolides ERYTH, VIRG-M1, VIRG-S1. The solid line is the median of the data points for that group.
